# Absence of the musculocutaneous nerve: a rare anatomical variation with possible clinical-surgical implications

**DOI:** 10.1590/S1516-31802008000500009

**Published:** 2008-09-04

**Authors:** José Humberto Tavares Guerreiro Fregnani, Maria Inez Marcondes Macéa, Celina Siqueira Barbosa Pereira, Mirna Duarte Barros, José Rafael Macéa

**Keywords:** Musculocutaneous nerve, Median nerve, Brachial plexus, Hypoesthesia, Paralysis, Nervo musculocutâneo, Nervo mediano, Plexo braquial, Hipoestesia, Paralisia

## Abstract

**CONTEXT:**

The musculocutaneous nerve is one of the terminal branches of the lateral fasciculus of the brachial plexus, and is responsible for innervation of the flexor musculature of the elbow and for skin sensitivity on the lateral surface of the forearm. Its absence has been described previously, but its real prevalence is unknown.

**CASE REPORT:**

A case of absence of the musculocutaneous nerve that was observed during the dissection of the right arm of a male cadaver is described. The area of innervation was supplied by the median nerve. From this, three branches emerged: one to the coracobrachialis muscle, another to the biceps brachii muscle and the third to the brachialis muscle. This last branch continued as a lateral antebrachial cutaneous nerve. This is an anatomical variation that has clinical-surgical implications, considering that injury to the median nerve in this case would have caused unexpected paralysis of the flexor musculature of the elbow and hypoesthesia of the lateral surface of the forearm.

## RESUMO

**CONTEXTO:**

O nervo musculocutâneo é um dos ramos terminais do fascículo lateral do plexo braquial, sendo responsável pela inervação da musculatura flexora do cotovelo e pela sensibilidade cutânea da face lateral do antebraço. Sua ausência já foi descrita previamente, mas a sua real prevalência é desconhecida.

**RELATO DE CASO:**

Este é um relato de caso da ausência do nervo musculocutâneo observada durante a dissecção do membro superior direito de um cadáver do sexo masculino, sendo o seu território de inervação suprido pelo nervo mediano. Deste emergiam três ramos, um para o músculo coracobraquial, outro para o músculo bíceps braquial e o terceiro para o músculo braquial. Este último ramo continuava-se como nervo cutâneo lateral do antebraço. Trata-se de variação anatômica que tem implicações clínico-cirúrgicas, já que a lesão do nervo mediano, neste caso, acarretaria inesperada paralisia da musculatura flexora do cotovelo e hipoestesia da face lateral do antebraço.

## INTRODUCTION

Contrary to what might be imagined, anatomical variations involving the brachial plexus are not very unusual: they are found in around 13% of dissections on cadavers. The most common of these variations involve abnormal positioning of the fasciculi in relation to the axillary artery, presence of anomalous communicating branches between the fasciculi, absence of the posterior fasciculus or, furthermore, unusual formation and paths for the median nerve.^1^

The musculocutaneous nerve is one of the terminal branches of the lateral fasciculus of the brachial plexus, and is responsible for innervation of the flexor musculature of the elbow and for skin sensitivity on the lateral surface of the forearm.^2^ Its absence has been described previously, but its real prevalence is unknown.^3,4^ This paper reports on a case of absence of the musculocutaneous nerve, in which its area of innervation was supplied by the median nerve.

## CASE REPORT

**Figure 1:**
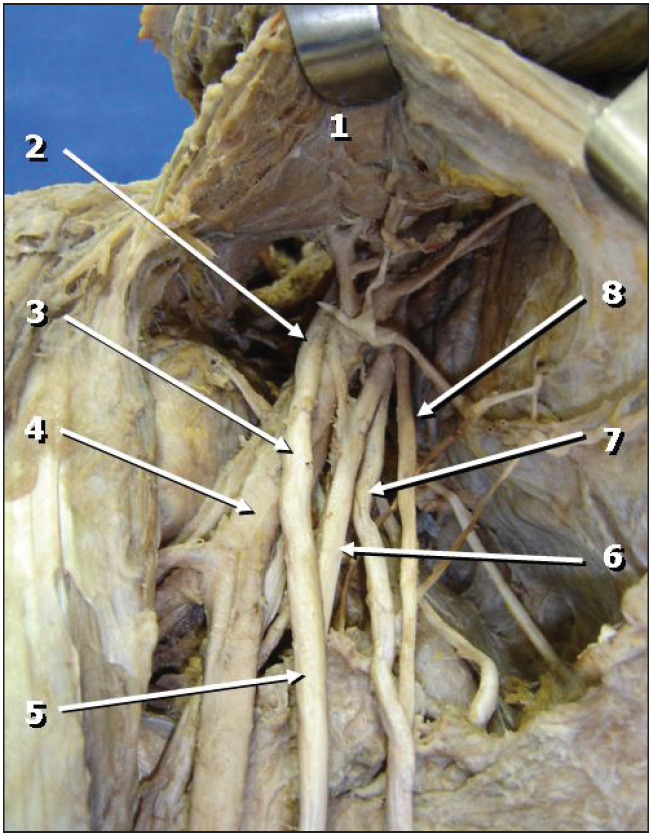
Lateral view of the right axilla. The lateral fasciculus of the brachial plexus (2) continues as the lateral root of the median nerve (3) without giving rise to the musculocutaneous nerve. Legend: 1) Pectoralis minor muscle; 2) Lateral fasciculus of the brachial plexus; 3) Lateral root of the median nerve (continuation of the lateral fasciculus); 4) Axillary artery; 5) Median nerve; 6) Medial root of the median nerve; 7) Ulnar nerve; 8) Medial antebrachial cutaneous nerve.

During macroscopic dissection of the right arm of a male cadaver, it was observed that the lateral fasciculus followed its usual path without giving rise to the musculocutaneous nerve, and continued as the lateral root of the median nerve (Figure 1). The only branch of the lateral fasciculus was the lateral pectoral nerve.

**Figure 2:**
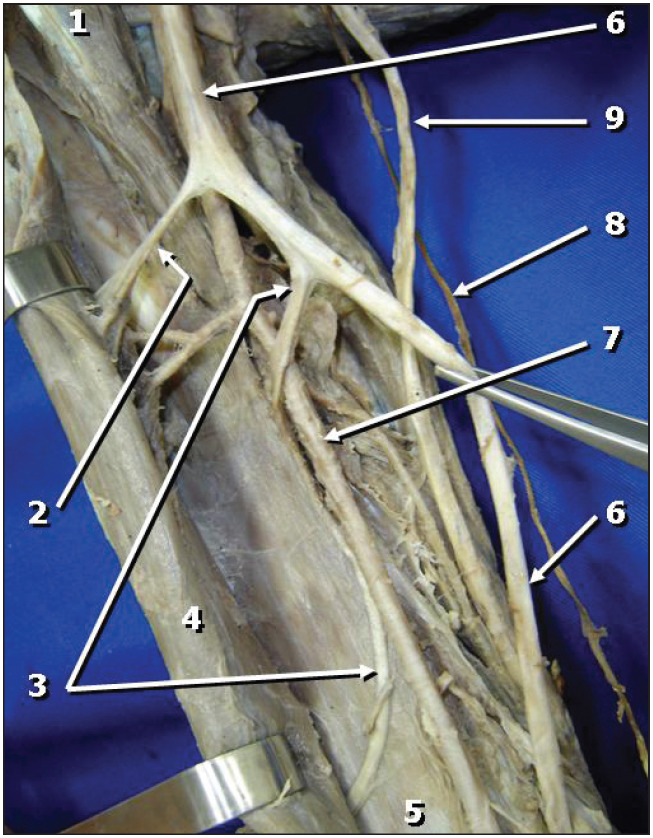
Anterior view of the right arm. Branches emerge from the lateral margin of the median nerve (2 and 3), heading towards the biceps brachii and brachialis muscles. Legend: 1) Coracobrachialis muscle; 2) Branch of the median nerve to the biceps brachii muscle; 3) Branch of the median nerve to the brachialis muscle; 4) Biceps brachii muscle; 5) Brachialis muscle; 6) Median nerve; 7) Brachial artery; 8) Medial antebrachial cutaneous nerve; 9) Ulnar nerve.

**Figure 3:**
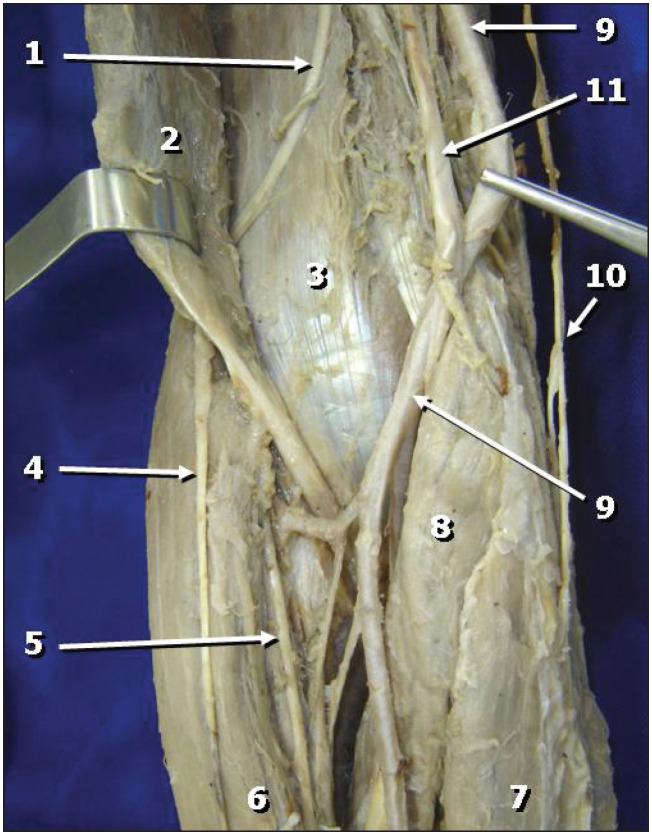
Anterior view of the right cubital fossa. The branch of the median nerve to the brachialis muscle (1) goes in deeply to the biceps brachii muscle and then continues as the lateral antebrachial cutaneous nerve (4). Legend: 1) Branch of the median nerve to the brachialis muscle; 2) Biceps brachii muscle; 3) Brachialis muscle; 4) Lateral antebrachial cutaneous nerve; 5) Superficial branch of the radial nerve; 6) Brachioradialis muscle; 7) Flexor carpi ulnaris muscle; 8) Pronator teres muscle; 9) Brachial artery; 10) Medial antebrachial cutaneous nerve; 11) Median nerve.

The biceps brachii, brachialis and coracobrachialis muscles were innervated by three branches that emerged from the median nerve in the arm. The first of these (the most cranial branch) was small and short and headed for the coracobrachialis muscle, branching out into small filaments. Because of its small size and fragility, it could not be preserved during the macroscopic dissection. The second branch emerged from the median nerve, at a point close to the caudal extremity of the coracobrachialis muscle, and headed laterally to the depth of the biceps brachii muscle, to innervate it (Figure 2). The third branch, which was the most distal and the longest, emerged around two centimeters below the exit of the second branch (Figure 2). After giving rise to branches to the brachialis muscle, it took a curvilinear lateral path between the biceps brachii and brachialis muscles, and came to the surface at the lateral margin of the forearm. It then followed a descending path over the brachioradialis muscle as the lateral antebrachial cutaneous nerve (Figure 3).

No other anatomical variations were found in the brachial plexus or the nerves of the arm, forearm and hand. Nor were there any in the subclavian, axillary or brachial arteries or their branches. The only other anatomical variation observed was in the cephalic vein, which was a tributary of the external jugular vein instead of forming its usual junction with the axillary vein.

## DISCUSSION

The musculocutaneous nerve is formed by motor-sensory fibers coming from the primary ventral branches of the C5 to C7 spinal nerves. After emerging from the lateral fasciculus, it heads towards the coracobrachialis muscle, which it penetrates, and continues deeply between the brachialis and biceps brachii muscles, to innervate all three of these muscles. Close to the cubital fossa, it comes to the surface laterally to the biceps brachii muscle and anteriorly to the brachialis muscle, and becomes known as the lateral antebrachial cutaneous nerve. This takes a descending path along the lateral margin of the forearm and leaves cutaneous branches to the lateral surface of the forearm.^2^

There is little data in the literature on the prevalence of absence of the musculocutaneous nerve. Beheiry dissected 60 arms and noted absence of the nerve in only one of them (1.7%).^4^ Prasada Rao and Chaudhary^3^ did not find this nerve in 8% of the 24 arms they dissected. Sometimes the absence of this nerve is only apparent. Nakatani et al. published a report on three cases in which the lateral fasciculus, median nerve and musculocutaneous nerve were wrapped in a single sheath of conjunctive tissue. After removal of this sheath, the musculocutaneous and median nerves were separated out.^5^

Absence of the musculocutaneous nerve does not lead to paralysis of the flexor musculature of the elbow and hypoesthesia of the lateral surface of the forearm, since the motor and sensitive fibers can arise from other nerves. The most common situation is that its fibers originate from the median nerve or, less frequently, from the lateral root of the median nerve or from the lateral fasciculus of the brachial plexus.^6-10^ Thus, this anatomical variation has no clinical manifestation and it is unlikely to be identified until a dysfunction of some of the nerves mentioned above appears.

The median nerve is formed by the confluence of the lateral and medial roots of the brachial plexus, which originate respectively from the lateral fasciculus (C5 to C7) and medial fasciculus (C8 and T1). Normally, it does not give rise to any branch in the axilla or arm, and is destined for motor innervation of almost all the muscles of the anterior compartment of the forearm (except for the flexor carpi ulnaris muscle and the medial half of the flexor digitorum profundus muscle) and some muscles of the hand (the muscles of the thenar eminence and the two most lateral lumbricalis muscles). It also sensitively innervates the skin of part of the palm, and part of fingers I, II and III and the lateral half of finger IV.^2^

It must be noted that the primary ventral branches of the spinal nerves that form the musculocutaneous nerve and the lateral root of the median nerve are common to these two nerves (from C5 to C7). Considering that in the present case the musculocutaneous nerve was absent, it is not a surprise that the nerve fibers heading for the flexor musculature of the elbow and the skin of the lateral surface of the forearm (coming from the C5 to C7 spinal nerves) would accompany those of the median nerve in the lateral fasciculus and, from there, would follow the median nerve along its path in the forearm.

This common origin of the median and musculocutaneous nerves also explains the frequent presence of communicating branches between these two nerves, which are found in up to one third of all individuals.^11^ Venieratos and Anagnostopoulou^12^ described three types of communication: type I, in which the communication is proximal to where the musculocutaneous nerve enters the coracobrachialis muscle; type II, in which the communication is distal to the point of entry into the muscle; and type III, in which the communication and the nerves do not penetrate the muscle. Type II is the most common (45.4%), followed sequentially by type I (41.0%) and type III (13.6%).

The anatomical variation described here has practical implications, since injury to the median nerve in the axilla or arm would, in this case, have caused unexpected paresis or paralysis of the flexor musculature of the elbow and hypoesthesia of the lateral surface of the forearm, in addition to the classical signs that are already well known. Injury to the median nerve could occur in cases of open or closed trauma to the arm, such as bullet and blade wounds. Iatrogenic injuries to the median nerve during surgery on the axilla or arm might also cause the clinical situation described above. The median nerve and its roots are close to the axillary vein, which is used as the most cranial limit for axillary lymph node dissection, a procedure that is used in treating certain tumors, such as breast carcinoma and melanoma. If the dissection extends more cranially than normal, injury to the median nerve (or to its medial root) may occur, with consequent dysfunction of the flexor musculature of the elbow if the anatomical variation described here is present. There could be similar occurrences during surgery on the arm if the surgeon believes that these are nerve branches of little importance and then he sections the branches of the median nerve that are heading for the flexor musculature of the elbow. It would not be unlikely for such accidents to occur even with the most eminent surgeons, considering that the classical concept is that the median nerve does not give rise to branches in the arm.
